# ProteomicsML: An Online Platform for Community-Curated Data sets and Tutorials for Machine Learning in Proteomics

**DOI:** 10.1021/acs.jproteome.2c00629

**Published:** 2023-01-24

**Authors:** Tobias G. Rehfeldt, Ralf Gabriels, Robbin Bouwmeester, Siegfried Gessulat, Benjamin A. Neely, Magnus Palmblad, Yasset Perez-Riverol, Tobias Schmidt, Juan Antonio Vizcaíno, Eric W. Deutsch

**Affiliations:** † Institute for Mathematics and Computer Science, University of Southern Denmark, 5000 Odense, Denmark; ‡ VIB-UGent Center for Medical Biotechnology, VIB, Ghent 9052, Belgium; § Department of Biomolecular Medicine, Ghent University, Ghent 9052, Belgium; ∥ MSAID Gmbh, Berlin 10559, Germany; ⊥ National Institute of Standards and Technology, Charleston, South Carolina 29412, United States; # Center for Proteomics and Metabolomics, Leiden University Medical Center, 2300 RC Leiden, The Netherlands; ⊗ European Molecular Biology Laboratory, European Bioinformatics Institute (EMBL-EBI), Wellcome Trust Genome Campus, Hinxton, Cambridge CB10 1SD, United Kingdom; ¶ MSAID GmbH, Garching b. Munich 85748, Germany; ∇ Institute for Systems Biology, Seattle, Washington 98109, United States

**Keywords:** machine learning, deep learning, proteomics, educational platform, community platform, bioinformatics

## Abstract

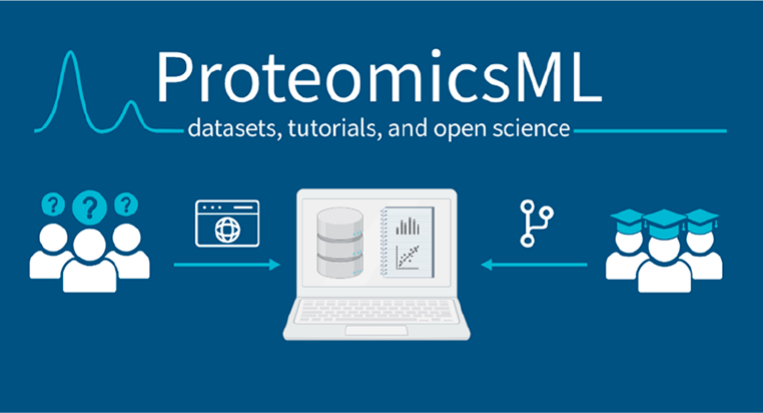

Data set acquisition and curation are often the most difficult and time-consuming parts of a machine learning endeavor. This is especially true for proteomics-based liquid chromatography (LC) coupled to mass spectrometry (MS) data sets, due to the high levels of data reduction that occur between raw data and machine learning-ready data. Since predictive proteomics is an emerging field, when predicting peptide behavior in LC-MS setups, each lab often uses unique and complex data processing pipelines in order to maximize performance, at the cost of accessibility and reproducibility. For this reason we introduce ProteomicsML, an online resource for proteomics-based data sets and tutorials across most of the currently explored physicochemical peptide properties. This community-driven resource makes it simple to access data in easy-to-process formats, and contains easy-to-follow tutorials that allow new users to interact with even the most advanced algorithms in the field. ProteomicsML provides data sets that are useful for comparing state-of-the-art machine learning algorithms, as well as providing introductory material for teachers and newcomers to the field alike. The platform is freely available at https://www.proteomicsml.org/, and we welcome the entire proteomics community to contribute to the project at https://github.com/ProteomicsML/ProteomicsML.

## Introduction

Computational predictions of analyte behavior in the context of mass spectrometry (MS) data have been explored for nearly five decades, with early rudimentary predictions dating back to 1983.^1^ With the rise of technology and computational power, machine learning (ML) approaches were introduced into the field of proteomics in 1998^2^ and ML-based models quickly overtook human accuracy. Since then, dozens of articles have described efforts to train models for a multitude of physicochemical properties associated with the field of high-throughput proteomics, as reviewed by Neely et al.^3^ Some of the most-commonly studied properties are retention time and fragmentation spectrum intensities, while a large range of lesser explored properties exists as well. For an exhaustive review of the current undertakings, see Wen et al. and Bouwmeester et al.^4,5^ While many of these efforts are still in the realm of basic exploratory research, ML approaches are increasingly being incorporated into mainstream tools and standalone predictive resources.^4,6−8^

When training any ML model, it is crucial to obtain suitable training and evaluation data sets. Likewise, in many fields of research where ML is applied, it is common to have a range of educational data sets, such as the MNIST (Modified National Institute of Standards and Technology)^9^ or IRIS (https://archive.ics.uci.edu/ml/datasets/iris) data sets, allowing newcomers to the field to easily learn common ML methodologies. Likewise, state-of-the-art models can use benchmark data sets such as ImageNet (https://www.image-net.org) or those available on the UCI Machine Learning Repository (https://archive.ics.uci.edu) to compare their predictive capabilities. Similar to the utility of benchmark data sets, such as the number of survivors on the Titanic, which has been modeled more than 54 000 times (https://www.kaggle.com/competitions/titanic), we seek to define proteomics data sets that can provide an entry point for ML modeling.

Although there have been numerous efforts to explore the predictive capabilities of models, there are barriers that limit widespread adoption in the field of predictive proteomics. First, there are considerable difficulties in accessing data sets in a suitable form for ML applications. A substantial effort is required to prepare raw proteomics data sets into a format usable for ML, as this demands extensive knowledge of the multitude of proteomics file formats and postprocessing methods. MS data also has a tendency to be fraught with missing metadata, making it challenging to compare across data sets. Furthermore, most ML frameworks in proteomics implement dedicated postprocessing pipelines to prepare the files for ML algorithms. Recently, tools such as ppx^10^ and MS2AI^11^ were created to facilitate this process, but they are still limited to certain use cases due to the complex nature of liquid chromatography coupled to mass spectrometry (LC-MS) data.

Second, while some ML-ready data sets are available on platforms such as Kaggle^12^ or in supplementary tables of publications, they are often difficult to find and lack long-term maintenance and support postpublication. While there is no formal consensus in the field, there are certain data sets that are often used for training such as ProteomeTools.^13^ Nevertheless, there are no widely used data sets used to compare the performance of tools developed by different researchers, making it difficult for new algorithms to be evaluated and compared to older tools. This issue is only further exacerbated by individual groups relying on different pre- and postprocessing protocols, such as differences in normalization of measurements or in the implementation of model performance metrics.

As an outcome of the 2022 Lorentz Center Workshop on Proteomics and Machine Learning (Leiden, The Netherlands, March 2022), we have created a web platform to facilitate the application of ML approaches to the field of MS-based proteomics. The resource is intended to provide a central focal point for curating and disseminating data sets that are ready to use for ML research, and to encourage new entrants into the field through expert-driven tutorials.

Here we describe how ProteomicsML has been developed using commonly available tools and designed for future ease of maintenance. We provide a brief overview of the data sets that are currently available at ProteomicsML and how it can be expanded in the future with more data. We also describe the initial set of tutorials that can be used as an introduction to the field of ML in proteomics.

## The ProteomicsML Platform

The primary entry point for the resource is the ProteomicsML Web site (https://www.proteomicsml.org/). It contains general introductory data sets that are already preprocessed and ready for training or evaluation, and contains educational resources in the form of tutorials for those new to ML in proteomics. The code base for the Web site is maintained via a GitHub repository (https://github.com/ProteomicsML/ProteomicsML), and is therefore easy to maintain and amenable to outside contributions from the community. On the GitHub repository, researchers can open pull requests (proposals for adding or changing information) for new data sets or tutorials. These pull requests are then reviewed by the maintainers, currently the authors of this paper, in line with the guidelines in the contributing section of the ProteomicsML Web site. Data sets and tutorials hosted as part of the GitHub repository fall under the CC BY 4.0 license, as indicated on both the repository and the Web site. The PRIDE database infrastructure^14^ is also used to store larger data sets on an FTP server dedicated to ProteomicsML.

A key goal of ProteomicsML is to advance with the field, which is why we provide a platform with detailed documentation, including a contributing guide on how to upload data sets and tutorials for specific ML workflows or algorithms. After curation by the maintainers, the contributions have to pass a build test in order to maintain integrity of the platform, and, if passed, are automatically published on the Web site and are freely accessible to other researchers.

For many LC-MS properties, such as retention time and fragmentation intensity, well-performing ML models have already been published. We aim to provide suitable data sets and tutorials to easily reproduce these results in an educational fashion. All data sets on the platform are organized by data type, and should ideally be provided in a simple data format that is suitable for direct import into ML toolkits. Each data type can contain one or more data sets for different purposes, and each data set should be sufficiently annotated with metadata (e.g., its origin, how it was processed, and the relevant literature citations).

Along with well-annotated data sets, the platform provides users with in-depth tutorials on how to download, import, handle, and train various ML models. Many of the LC-MS data types require certain, sometimes complex, preprocessing steps in order to be fully compatible with ML frameworks. For this reason, we believe it is crucial to provide guidelines on these processes to ultimately lower the entry barriers for new users to the field. Tutorials on ProteomicsML can be attribute- or data set-specific, allowing new tutorial submissions to focus on either the direct interactions with specific ML models or methodologies, or on a certain aspect of data preprocessing.

Often when new modeling approaches are published, they are accompanied by data sets with novel pre- and postprocessing steps. Using ProteomicsML, the new data can be uploaded to the site along with a unified metadata entry and an accompanying tutorial that improves reproducibility of the work and facilitates benchmarking by the community.

## Data Sets and Tutorials

The original raw data for proteomics data sets currently included in ProteomicsML have already been made publicly available through ProteomeXchange,^15^ mostly via the PRIDE database.^14^ Here, the data hosted at ProteomicsML are provided in an ML-ready format, with links to original metadata and raw files for full provenance. Even though the data sets at ProteomicsML do not contain raw files, we do provide users with extensive tutorials on how to process raw data into ML-ready formats. ProteomicsML currently contains data sets and tutorials for fragmentation intensity, ion mobility (IM), retention time, and protein detectability. More data types can easily be added in the future, as the platform evolves along with the field.

(1) Retention time. Due to retention time playing a major role in modern peptide identification workflows, it is one of the most explored properties in predictive proteomics.^4^ While some data sets for predicting retention time already exists, such as the publicly available data set from Kaggle (https://www.kaggle.com/datasets/kirillpe/proteomics-retention-time-prediction) and the DLOmix data sets (https://github.com/wilhelm-lab/dlomix/), we have also compiled new multitiered ML-ready data sets from the ProteomeTools synthetic peptide library,^13^ in three specific sizes: 100 000 data points (small), well suited for new practitioners; (ii) 250 000 data points (medium), and (iii) 1 million data points (large), well suited for larger-scale ML training or benchmarking. As amino acid modifications can complicate the application of ML in proteomics, these three tiers do not contain any modified peptides except for carbamidomethylation of cysteine. Nevertheless, to train models for more real-life applications, we have also included an additional data set tier containing 200 000 oxidized peptides, as well as a mixed data set containing 200 000 oxidized and 200 000 unmodified peptides. These data sets require minimal data preparation, although we still provide two distinct tutorials on methods to incorporate these data sets into deep learning (DL)-based models. In addition to preprocessed data, we also provide a detailed tutorial that combines and aligns retention times between runs from MaxQuant evidence files.^16^ The output of this tutorial is a fully ML-ready file for retention time prediction.
(2) Fragmentation intensity. While it is easy to calculate the *m*/*z* values of theoretical peptide spectra, fragment ion peak intensities follow complex patterns that can be hard to predict. Nevertheless, these intensities can play a key role in accurate peptide identification.^17^ For this reason, fragment ion intensity prediction is likely the second most explored topic for prediction purposes, for which comprehensive data sets and tutorials exist within ProteomicsML. As there are many attributes of peptides that affect their fragmentation patterns, the preprocessing steps of fragmentation data are more complex, and can be substantially different from lab to lab. For this reason, we have composed two separate tutorials, one that mimics the Prosit^6^ data processing approach on the ProteomeTools^13^ data sets, which consists of 745 000 annotated spectra, and one that mimics the MS^2^PIP data process on a consensus human spectral library from the National Institute of Standards and Technology, which consists of 270 440 annotated spectra.^18^ For data sets in this category it is difficult to provide a simple format with unified columns, as the handling and preprocessing steps differ significantly from model to model. Currently, there is one tutorial available on ProteomicsML describing the data processing pipeline from raw file to Prosit-style annotation, and we believe that with future additions we can provide users with tutorials for additional processing approaches.
(3) Ion mobility. Ion mobility is a technique to separate ionized analytes based on their size, shape, and physicochemical properties.^19^ Techniques for ion mobility are generally based on propelling or trapping ions with an electric field in an ion mobility cell. Peptides are then separated by colliding them with an inert gas without fragmentation. Indeed, peptides with a larger area to collide will be more affected by the collisions, resulting in a higher measured collisional cross section (CCS). Historically, most methods predicting ion mobility were based on molecular dynamics models that calculate the CCS from first-principles in physics.^20^ Lately the field has generated multiple ML and DL approaches for both peptide and metabolite CCS prediction.^21−23^ The tutorials made available in ProteomicsML use both trapping (trapped ion mobility,^24^ TIMS) and propelling ion mobility (traveling wave ion mobility,^25^ TWIMS) data, where the large TIMS data set was sourced from Meier et al.^23^ (718 917 data points) and the TWIMS data was sourced from Puyvelde et al.^26^ (6268 data points). The tutorial is a walkthrough for training various model types, ranging from simple linear models to more complex nonlinear models (e.g., DL-based networks) showing advantages and disadvantages of various learning algorithms for CCS prediction.
(4) Protein detectability. Modern proteomics methods and instrumentation are now routinely detecting and quantifying the majority of proteins thought to be encoded by the genome of a given species.^27^ Yet even after gathering enormous amounts of data, there is always a subset of proteins that remains refractory to detection. For example, even though tremendous effort has been focused on the human proteome, the fraction of unobserved proteins has been pushed just below 10%.^28,29^ It remains unclear why certain proteins remain undetected, although ML has been applied to explore which properties most strongly influence detectability (as reviewed within).^30^ One can compute a set of properties for a proteome and then train a model using those properties based on real world observations of the proteins that are detected and the proteins that are not detected. The model can be trained to learn which properties separate the detected from the undetected. Such a model has further utility to highlight proteins with properties that should sort them into the detected group, yet are not, as well as proteins that should belong to the undetected group, and yet they are detected. To facilitate this we have included the *Arabidopsis* PeptideAtlas data set (http://www.peptideatlas.org/builds/arabidopsis/), which is based on an extensive study of a single proteome.^31^ This data set is based on the 2021 build, which has 52 data sets reprocessed to yield 40 million peptide-spectrum matches and a good overall coverage of the *Arabidopsis thaliana* proteome. Proteins in the data set are categorized as either “canonical”, having the strongest evidence of detection, or “not observed”, for which no peptides are identified. Along with these class labels, the data set contains various protein properties such as molecular weight, hydrophobicity, and isoelectric point, which could be crucial for classification purposes. The data set has an accompanying tutorial that illustrates how to analyze the data with a classification model for the observability of peptides.

Overall, these initial data set submissions and tutorials leave room for future expansion, until the community resource contains data sets for all properties previously and currently being explored in the field of proteomics. It is also open for user submissions, allowing researchers to upload their data in a standardized fashion, along with in-depth tutorials on their data handling and ML methodologies, resulting in more reproducible science. Our expectation is that this will shape the future of predictive proteomics, in favor of being more accessible, standardized, and reproducible.

Additionally, we have compiled a list of proteomics publications that utilize ML, along with a list of ProteomeXchange data sets used by each of the publications (Supplementary Table 1). Each of these ProteomeXchange data sets have been given a set of tags to indicate the nature of the usage in the publications (e.g., benchmarking, retention time, deep learning, etc.) as shown in Supplementary Table 2 (https://github.com/PRIDE-Utilities/pride-ontology/blob/master/pride-annotations/projects-proteomicsML.csv). Furthermore, these tags have also been added to the respective PRIDE data sets, which allows the tags to be easily searched, and for users to compile their ideal data set, if ProteomicsML does not already contain one.

## Conclusion

We have presented ProteomicsML, a comprehensive resource of data sets and tutorials for every ML practitioner in the field of MS-based proteomics. ProteomicsML contains multiple data sets on a range of LC-MS peptide properties, allowing computational proteomics researchers to compare new algorithms to state-of-the-art models, as well as providing newcomers to the field with an accessible starting point, without requiring immediate in-depth knowledge of the entire proteomics analysis pipeline. We believe that this resource will aid the next generation of ML practitioners, and provide a stepping stone for more open and more reproducible science in the field.

## Supporting Information Available

Supplementary Table 1: Proteomics ML publications along with links to the ProteomeXchange data sets used for training or testing (XLSX)
Supplementary Table 2: Public ProteomeXchange data sets that have been used for ML training or benchmarking (XLSX)

## Notes

Identification of certain commercial equipment, instruments, software, or materials does not imply recommendation or endorsement by the National Institute of Standards and Technology (NIST), nor does it imply that the products identified are necessarily the best available for the proposed purpose.
